# Electropolymerized PAA as a Functional Matrix for CeO_2_-NiO Hybrid Electrocatalysts for Efficient Water Oxidation

**DOI:** 10.3390/polym17192631

**Published:** 2025-09-28

**Authors:** Mrunal Bhosale, Pritam J. Morankar, Yeonsu Lee, Hajin Seo, Chan-Wook Jeon

**Affiliations:** School of Chemical Engineering, Yeungnam University, 280 Daehak-ro, Gyeongsan 38541, Republic of Korea; mrunal.snst.1@gmail.com (M.B.); pritam.nanoworld@gmail.com (P.J.M.); leeyeonsu@yu.ac.kr (Y.L.); 22546098@yu.ac.kr (H.S.)

**Keywords:** electrochemical water splitting, electrocatalyst, CeO_2_-NiO-PAA/NF, ECSA

## Abstract

Electrochemical water splitting has emerged as a pivotal strategy for advancing sustainable and renewable energy technologies. However, its practical deployment is often hampered by sluggish reaction kinetics, large overpotentials, and the high cost of efficient electrocatalysts. To overcome these critical challenges, a novel bifunctional electrocatalyst based on electropolymerized CeO_2_-NiO with polyacrylic acid (Ce-Ni-PAA) has been rationally engineered for overall water splitting. The strategic incorporation of conductive polymer framework enables effective modulation of the local electronic structure, enhances charge transport pathways, and maximizes the density of electrochemically accessible active sites, thereby substantially boosting catalytic performance. When evaluated in a 1 M KOH alkaline medium, the optimized Ce-Ni-PAA_0.5_/NF hybrid demonstrates remarkable catalytic activity with 366.5 mV overpotential at 50 mA cm^−2^, coupled with lower Tafel slope of 93.5 mV dec^−1^. Additionally, the Ce-Ni-PAA_0.5_/NF electrocatalyst exhibits exceptional ECSA of 1092.3 cm^2^, which confirms the presence of a significantly larger number of electrochemically active sites. The electrocatalyst retains its performance even after 5000 continuous cycles of operation. The superior performance is attributed to the synergistic effects arising from the enriched composition, efficient electron transport channels, and abundant catalytic centers. Collectively, this study not only highlights the significance of rational structural and compositional design but also offers valuable insights toward the development of next-generation, cost-effective bifunctional electrocatalysts with strong potential for scalable water splitting and clean energy applications.

## 1. Introduction

Hydrogen generation through electrochemical water splitting, along with the advancement of fuel cells and metal-air batteries, is regarded as a viable strategy to mitigate pressing environmental and energy concerns. A critical step in these energy conversion systems is the oxygen evolution reaction (OER), which proceeds through a complex four-electron pathway and suffers from inherently slow kinetics [1,2,3,4]. Designing efficient, low-cost, and durable OER catalysts is vital to enhance performance and to enable the large-scale implementation of these emerging clean energy technologies [5,6,7]. Noble-metal based materials (e.g., Ru, Ir, Pt) are considered efficient OER catalysts; nevertheless, their scarcity and expensive cost restrict their extensive use [8,9,10]. In recent years, numerous low-cost OER electrocatalysts have been designed and explored with particular attention to transition metals such as Fe, Co, Ni, and Mn. These catalysts exhibit remarkable performance in alkaline electrolytes, including low onset potentials, high activity, and strong durability [11]. Due to their low overpotential, abundant active sites, and excellent corrosion resistance in alkaline electrolytes, Ni-based catalysts have been extensively reported as highly active materials for the OER. They are widely regarded as the most promising candidates among non-precious-metal-based electrocatalysts. In addition, the relatively low cost and natural abundance of nickel make these catalysts highly attractive for large-scale and sustainable water-splitting technologies [12,13,14,15]. Self-supported electrodes can be fabricated through various synthesis methods including hydrothermal or solvothermal routes, vapor deposition techniques, and electrodeposition processes. Among them, electrodeposition is a versatile and widely employed electrochemical technique for synthesizing thin films, nanostructures, and composite materials on conductive substrates. It involves the reduction or oxidation of metal ions in an electrolyte solution, driven by the application of an external electrical current or potential, resulting in the controlled deposition of metals, metal oxides, or alloys onto the electrode surface [16,17,18]. For instance, Manickam Minakshi et al., using micelle-assisted electrodeposited γ-MnO_2_, showed a specific capacitance exceeding 478.6 F g^−1^, doubling that of pristine MnO_2_, which is attributed to the improved morphology and increased electroactive surface area [19]. In another study, Manickam Minakshi et al. showed that biowaste-derived CaO electrodes achieve 47.5 F g^−1^ specific capacitance with nearly 100% retention after 1000 cycles [20]. Biswal, A. et al. demonstrated electrodeposited Co-Ni-Cu ternary oxides achieving specific capacitances of 188 F g^−1^ with 95.1% retention after 1000 cycles when modified with DTAB surfactant [21].

In addition, certain rare-earth compounds have attracted considerable attention as potential catalytic materials owing to their exceptional oxygen and hydrogen storage capacities, making them some of the most promising candidates among existing solid-state catalysts [22]. Among these, cerium oxide (CeO_2_) stands out as one of the most fundamental and widely studied rare-earth oxides [23]. Its significance arises from the facile and reversible redox transition between Ce^4+^ and Ce^3+^, which enables efficient oxygen storage and release during oxidation-reduction cycles [24]. This unique property not only enhances catalytic activity but also contributes to improved stability and durability in various energy conversion and environmental applications. For instance, 20-NiO@CeO_2_ appeared as an efficient electrocatalyst with a very low overpotential of 392 mV to reach 50 mA cm^−2^ current density [25]. A few studies have been reported on the electrocatalytic activity of CeO_2_/Ni/NC [24], CeO_2_/CuO [26] for OER. According to the volcano relationship model, NiO exhibits remarkable catalytic activity for the OER, as evidenced by its position near the apex of the OER volcano plot, indicating optimal binding energies for reaction intermediates [27]. The presence of Ni-O species in nickel oxide catalysts plays a pivotal role in enhancing electrocatalytic efficiency by facilitating rapid charge transfer and lowering the adsorption energy barrier for the OER intermediate, thereby accelerating the overall OER kinetics [28,29]. Recent advancements have reported the fabrication of ternary Cu-Co-Ni metal oxide systems supported on Ni foam, which demonstrated an overpotential of approximately 411 mV at a current density of 50 mA cm^−2^ in 1 M KOH electrolyte [30]. Furthermore, the incorporation of single-atom Pt into the crystalline lattice of porous NiO nanocubes (0.5 wt% Pt/NiO) has been shown to significantly boost catalytic performance, achieving a reduced overpotential of 400 mV at the same current density [31]. Incorporating metals, nonmetals, or polymers such as polyacrylic acid (PAA), chitosan, and starch has been shown to significantly improve the electrochemical activity of the catalyst [32,33]. The PAA is particularly attractive as it is a low-cost, biodegradable, and non-toxic synthetic anionic polymer with superabsorbent properties, composed of high molecular weight chains formed from acrylic acid monomers [34,35]. PAA, containing ionizable carboxyl (-COOH) groups responsive to pH and ionic strength, serves as an efficient protective capping polymer. Its ability to form stable complexes with metals, combined with hydrophilicity, selectivity, and mechanical robustness, enables it to improve electrochemical activity of catalyst [36,37]. The synthesis of mesoporous metal sulfide hybrids, specifically meso-Fe-MoS_2_/CoMo_2_S_4_, has been accomplished via a soft-templating strategy that employs the diblock copolymer polystyrene-block-poly (acrylic acid) (PS-b-PAA) micelles as structure-directing agents. The resultant meso-Fe-MoS_2_/CoMo_2_S_4_ demonstrates outstanding electrocatalytic performance for the OER, exhibiting an overpotential of 290 mV at a current density of 10 mA cm^−2^ [37]. Furthermore, investigations into the influence of PAA concentration have revealed its pivotal role in catalytic activity of hollow bimetallic mixed oxide spheres based on nickel and molybdenum. Optimized hollow Mo-Ni oxide microspheres, fabricated with tailored PAA concentrations, achieve a competitive OER overpotential of 330 mV at 10 mA cm^−2^ [38].

Although several studies have reported CeO_2_-NiO composites for electrocatalysis, most rely on multi-step synthesis routes and do not address the simultaneous control of morphology, surface defects, and interfacial contact in a single, facile step. Furthermore, the role of polymer additives in previous studies has generally been limited to acting as simple binders or dispersants, with little systematic investigation of their effect on electronic structure or catalytic kinetics. In this work, a facile electrodeposition strategy was employed to synthesize CeO_2_-NiO-PAA (Ce-Ni-PAA/NF) nanocomposites directly on nickel foam (NF), enabling uniform deposition and intimate interfacial contact. Comprehensive spectroscopic and structural characterizations confirmed the successful formation of the Ce-Ni-PAA/NF hybrid catalyst, revealing NiO nanoparticles homogeneously anchored on the surface of CeO_2_ nanoflakes, thereby generating a hierarchical architecture with abundant electroactive sites. Among the various electrodeposited compositions, Ce-Ni-PAA_0.5_/NF exhibited superior OER performance, delivering a low overpotential of 366.5 mV at 50 mA cm^−2^ along with a favorable Tafel slope, indicative of fast reaction kinetics. The remarkable activity can be ascribed to the synergistic interplay between CeO_2_ and NiO, which facilitates charge transfer, enhances the electrochemically active surface area, and optimizes the adsorption/desorption of OER intermediates. Furthermore, the catalyst demonstrated excellent electrochemical robustness, as confirmed by prolonged cyclic voltammetry (CV) cycling and chronopotentiometry measurements, affirming its structural integrity and operational stability under harsh alkaline conditions.

## 2. Experimental Section

### 2.1. Chemicals

Cerium nitrate hexahydrate (Ce(NO_3_)_3_·6H_2_O) was obtained from Alfa Aesar, Gangnam-gu, Seoul Republic of Korea, while nickel nitrate hexahydrate (Ni(NO_3_)_2_·6H_2_O) and potassium nitrate (KNO_3_) were sourced from Sigma-Aldrich, St. Louis, MO, USA. Potassium hydroxide (KOH, ≥85%) was supplied by DaeJung Chemicals & Metals Co., Gyeonggi-do, Republic of Korea. High-purity nickel foam, serving as the conductive substrate, was procured from NARA Cell-Tech Corporation, Seoul, Republic of Korea. All reagents were utilized as received without any additional purification, and deionized (DI) water was consistently employed in all synthesis and electrochemical experiments to maintain reproducibility and minimize contamination.

### 2.2. Synthesis of CeO_2_, CeO_2_-NiO, and CeO_2_-NiO-PAA Precursor Solution

In this study, the precursor solution for CeO_2_ was prepared by dissolving 0.217 g of Ce(NO_3_)_3_·6H_2_O and 0.201 g of KNO_3_ in 100 mL of DI water under continuous magnetic stirring for 30 min to ensure complete dissolution and homogeneity. For the synthesis of the CeO_2_–NiO binary composite, 0.108 g of Ni(NO_3_)_2_·6H_2_O was subsequently introduced into the CeO_2_ precursor solution and stirred for 30 min. To obtain the ternary CeO_2_-NiO-PAA composite, PAA was incorporated into the Ce–Ni solution at three different concentrations (0.2, 0.5, and 0.8 g), yielding precursor mixtures with varied polymer contents.

**Figure 1:**
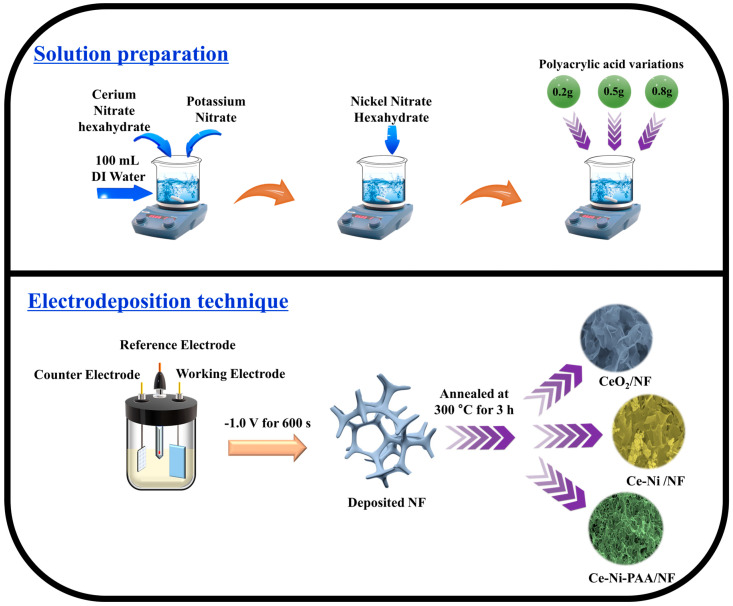
Schematic representation of electrocatalysts synthesis.

The resulting solutions were utilized for direct electrodeposition of CeO_2_, CeO_2_-NiO, and CeO_2_-NiO-PAA composites onto pre-cleaned nickel foam substrates. Electrodeposition was performed via chronoamperometry at a constant potential of −1.0 V for 600 s to ensure uniform film growth. The as-deposited electrodes were designated as Ce-Ni/NF, Ce-Ni-PAA_0.2_/NF, Ce-Ni-PAA_0.5_/NF, and Ce-Ni-PAA_0.8_/NF, corresponding to the different PAA loadings. After deposition, the electrodes were thoroughly rinsed with DI water and subsequently annealed at 300 °C for 3 h to improve crystallinity and interfacial stability. A schematic representation of the synthesis and deposition process is illustrated in Figure 1.

### 2.3. Material Characterization

The crystallographic structure of the synthesized nanomaterials was analyzed using an X-ray diffractometer (X’Pert Pro, PANalytical, Almelo, The Netherlands) equipped with a Cu Kα radiation source (λ = 1.5406 Å), enabling precise phase identification. The surface chemical composition and oxidation states of the constituent elements were probed by X-ray photoelectron spectroscopy (XPS) on a Thermo Scientific K-Alpha spectrometer, Cheshire, UK analysis system, providing insight into electronic structure and surface chemistry. Furthermore, the morphological features, particle size distribution, and textural characteristics were examined using high-resolution scanning electron microscopy (SEM, HITACHI S-4800, Tokyo, Japan) coupled with an energy-dispersive X-ray spectroscopy (EDX) system, allowing detailed elemental composition analysis and spatially resolved elemental mapping

### 2.4. Electrochemical Analysis

Electrochemical measurements were performed using a Biologic Instrument WBCS3000 battery cycler (WonATech Co., Ltd., Seoul, Republic of Korea) in a conventional three-electrode configuration. The catalyst-modified NF served as the working electrode, while a Hg/HgO electrode and a platinum plate were employed as the reference and counter electrodes, respectively. All experiments were conducted in a nitrogen-saturated 1 M KOH electrolyte. Cyclic voltammetry (CV) was recorded in the non-Faradaic region over a potential window of 0.1–0.2 V at scan rates of 5, 10, 15, 20, and 25 mV s^−1^ to evaluate the electrochemical double-layer capacitance (C_dl_) and estimate the electrochemically active surface area (ECSA). Linear sweep voltammetry (LSV) was carried out at a scan rate of 5 mV s^−1^ within the potential range of 0 to 1 V to determine the overpotential associated with the OER. All potentials measured versus Hg/HgO were converted to the reversible hydrogen electrode (RHE) scale using the Nernst equation:

E_RHE_ = E_Hg/HgO_ + E°_Hg/HgO_ + 0.0591 × (pH) (1)

where E°_Hg/HgO_ is the standard potential of the Hg/HgO reference electrode, and the pH of 1 M KOH is approximately 13.9. The long-term durability of the optimized electrocatalyst was assessed through LSV measurements before and after 5000 CV cycles, as well as by chronopotentiometry at a constant current density, thereby confirming structural stability and catalytic robustness under prolonged OER conditions.

## 3. Result and Discussion

**Figure 2:**
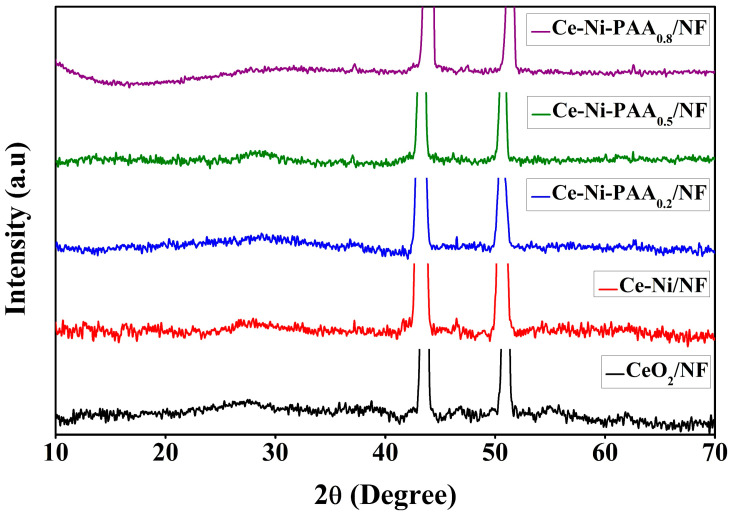
XRD spectra of all the composite.

The crystallographic structure, phase purity, and structural integrity of the synthesized materials were comprehensively analyzed by X-ray diffraction (XRD), as depicted in Figure 2. All diffraction patterns were recorded at room temperature over a 2θ range of 10–70°. The pristine CeO_2_ sample displayed well-defined diffraction peaks at 28.2°, 47.1°, and 55.7°, corresponding to the (111), (220), and (311) planes, respectively, which are in excellent agreement with the standard cubic fluorite structure of CeO_2_ (JCPDS card No. 00-034-0394), thereby confirming the successful formation of phase-pure CeO_2_ [39,40]. In addition to the characteristic CeO_2_ peaks, reflections at 44.2° and 51.1° were attributed to the nickel foam substrate. For the Ce–Ni composite, distinct diffraction peaks at 37.2°, 43.2°, and 62.5° were indexed to the (101), (012), and (110) planes of NiO, confirming the formation of the rhombohedral NiO phase in good agreement with JCPDS card No. 01-044-1159 [41,42]. Notably, after the introduction of NiO, the nickel foam reflections exhibited a slight shift toward lower diffraction angles, further validating the successful growth and strong interaction of NiO on the NF substrate. Interestingly, following the incorporation of PAA, a slight shift in the diffraction peaks was observed, indicating enhanced interfacial interaction between CeO_2_ and NiO. This shift suggests a more compact lattice structure, which may contribute to improved charge transfer dynamics and synergistic catalytic activity.

**Figure 3:**
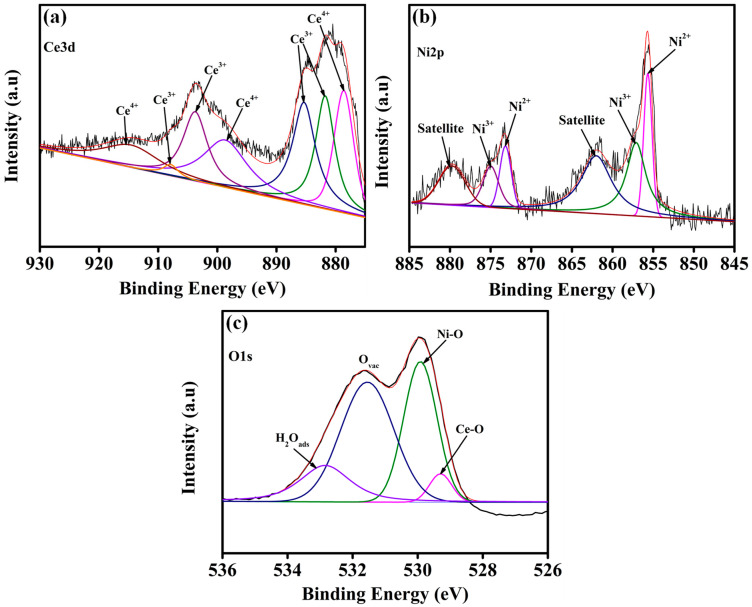
High resolution XPS spectra of (**a**) Ce3d, (**b**) Ni2p, and (**c**) O1s of Ce-Ni-PAA_0.5_/NF composite.

The chemical composition and oxidation states of Ce-Ni-PAA_0.5_/NF were systematically investigated by X-ray photoelectron spectroscopy (XPS), as illustrated in Figure 3. The high-resolution Ce3d spectrum (Figure 3a) was deconvoluted into seven well-resolved peaks, revealing the coexistence of Ce^4+^ and Ce^3+^ oxidation states. The prominent peaks located at binding energies of 878.7, 898.6, 907.8, and 916.0 eV correspond to the Ce^4+^ 3d_5/2_ and 3d_3/2_ spin–orbit components, confirming the presence of the fully oxidized CeO_2_ phase. In contrast, the peaks at 881.8, 885.3, and 903.6 eV are assigned to the Ce^3+^ 3d_5/2_ and 3d_3/2_ states, indicating partial reduction of Ce^4+^ to Ce^3+^ [43,44,45]. The deconvoluted Ni2p spectrum (Figure 3b) exhibited two distinct spin–orbit doublets, characteristic of mixed-valence Ni species, accompanied by two well-defined satellite peaks. The peaks at 855.6 and 873.1 eV are attributed to Ni^2+^ (2p_3/2_ and 2p_1/2_), while those at 856.9 and 874.9 eV confirm the presence of Ni^3+^ species, suggesting a partially oxidized NiO phase with enhanced redox activity. The shake-up satellite peaks appearing at ~860.9 and 879.5 eV further support the presence of Ni^2+^ in the system [46,47,48]. The O1s core-level spectrum (Figure 3c) was deconvoluted into four components at 529.0, 529.8, 531.5, and 532.8 eV, corresponding to lattice oxygen in Ce-O bonds, Ni-O bonds, oxygen vacancy-related species (O_Vac_), and surface-adsorbed molecular water (H_2_O_ads_), respectively. The significant contribution of oxygen vacancy peaks highlights the defect-rich nature of the Ce-Ni-PAA_0.5_/NF surface, which is expected to facilitate enhanced charge transfer and promote OER kinetics [43,48].

**Figure 4:**
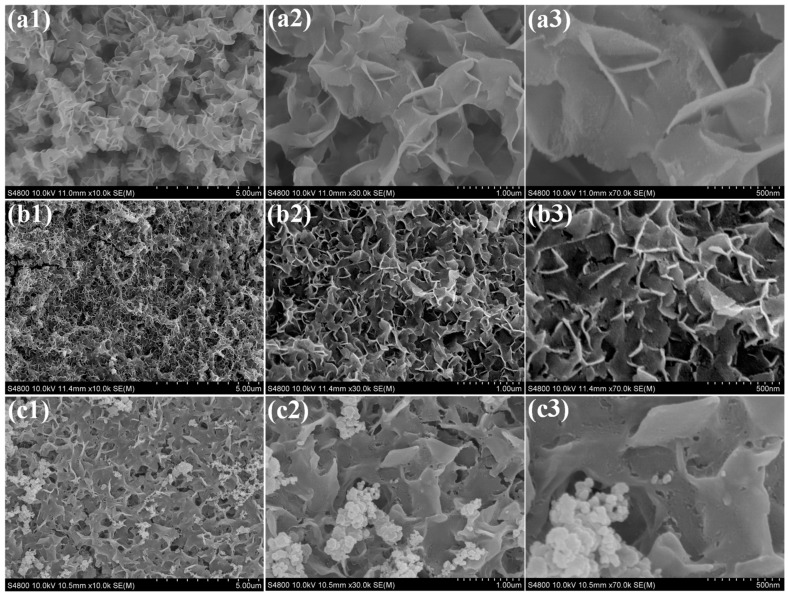
SEM images of (**a1**–**a3**) CeO_2_/NF, (**b1**–**b3**) Ce-Ni/NF, and (**c1**–**c3**) Ce-Ni-PAA_0.5_/NF.

The surface morphology and microstructural features of the synthesized electrocatalysts were systematically investigated by scanning electron microscopy (SEM), as presented in Figure 4. The SEM micrographs of pristine CeO_2_ (Figure 4(a1–a3)) reveal a well-defined nanoflake-like architecture, homogeneously distributed across the nickel foam substrate, forming an interconnected network with high surface coverage. In the Ce-Ni binary composite (Figure 4(b1–b3)), NiO nanoparticles are observed to be uniformly anchored onto the CeO_2_ nanoflakes, forming a hierarchical nanostructure. The NiO particles exhibit slightly irregular morphologies but maintain intimate contact with the underlying CeO_2_, suggesting strong interfacial interaction, which is beneficial for charge transfer and electrocatalytic performance. For the Ce-Ni-PAA ternary composites, PAA functions as a structure-directing agent, significantly influencing the surface morphology. In Ce-Ni-PAA_0.2_ and Ce–Ni–PAA_0.5_ as shown in Figure S1(a1–a3) and Figure 4(c1–c3), the CeO_2_ nanoflakes appear noticeably thicker compared to the pristine CeO_2_ and Ce-Ni composites, and the NiO nanoparticles exhibit an increased size distribution, indicating enhanced growth in the presence of PAA. This structural thickening likely contributes to improved mechanical stability and a higher electrochemically active surface area. However, in the Ce-Ni-PAA_0.8_ composite (Figure S1(b1–b3)), excessive PAA loading leads to partial agglomeration of nanostructures, which may result in reduced surface exposure of active sites and hindered mass transport. This observation suggests that an optimal PAA concentration is crucial to achieving a balance between structural stability and accessible catalytic sites.

**Figure 5:**
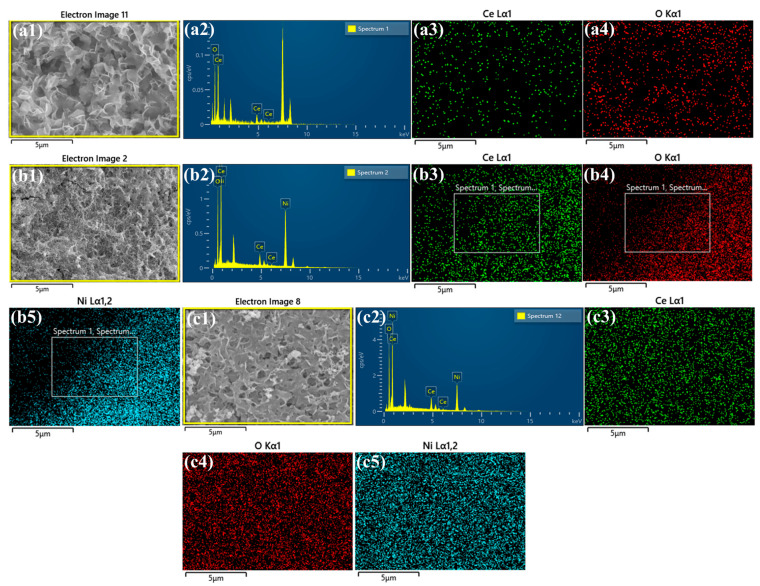
EDAX analysis data of (**a1**,**a2**) CeO_2_/NF, (**b1**,**b2**) Ce-Ni/NF, and (**c1**,**c2**) Ce-Ni-PAA_0.5_/NF. Elemental mapping data of (**a3**,**a4**) CeO_2_/NF, (**b3**–**b5**) Ce-Ni/NF, and (**c3**–**c5**) Ce-Ni-PAA_0.5_/NF.

The elemental composition and spatial homogeneity of the synthesized electrocatalysts were thoroughly examined by energy-dispersive X-ray spectroscopy (EDX). For the pristine CeO_2_/NF sample (Figure 5(a1,a2)), the composition was found to consist of 47.68 wt% Ce and 52.32 wt% O, confirming the formation of stoichiometric CeO_2_. In the Ce-Ni/NF composite (Figure 5(b1,b2)), the elemental distribution revealed 11.09 wt% Ce, 15.43 wt% O, and a significantly higher Ni content of 73.48 wt%, which can be attributed to both the successful incorporation of NiO. For the ternary Ce-Ni-PAA_0.5_/NF composite (Figure 5(c1,c2)), the composition showed 20.05 wt% Ce, 21.15 wt% O, and 58.80 wt% Ni, indicating a well-balanced integration of all three constituents. Elemental mapping provided further insights into the spatial distribution of these elements. As depicted in Figure 5(a3,a4), Figure 5(b3–b5), and Figure 5(c3–c5), Ce, O, and Ni were homogeneously distributed across the CeO_2_/NF, Ce-Ni/NF, and Ce-Ni-PAA_0.5_/NF samples, respectively, confirming uniform deposition and intimate interfacial contact. Similar results for Ce-Ni-PAA_0.2_/NF and Ce-Ni-AA_0.8_/NF are presented in Figures S2(a1–a5) and S3(b1–b5), showing consistent elemental dispersion. Such a uniform distribution of active species is critical for ensuring a high density of electrochemically accessible sites, efficient charge transport, and enhanced catalytic activity toward the OER.

**Figure 6:**
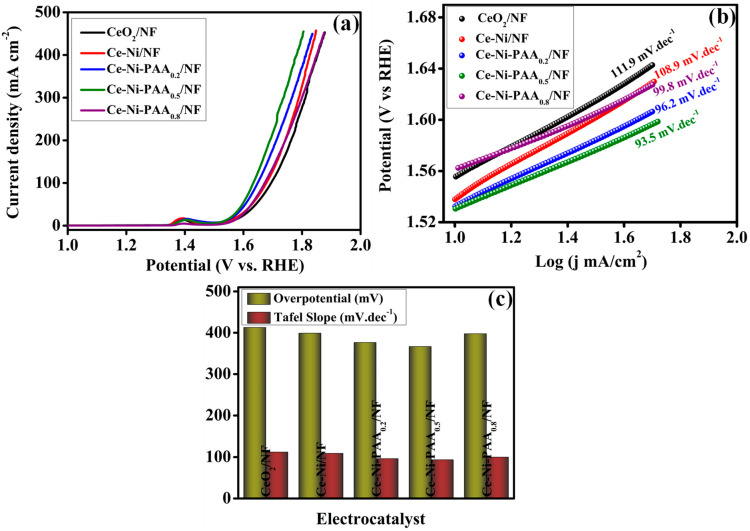
Electrochemical characterizations of electrocatalysts OER performances: (**a**) LSV curves at 5 mV s^−1^ scan rate, (**b**) Tafel slopes, and (**c**) evaluation of the OER performance concerning overpotential at 50 mA/cm^2^ and Tafel slope.

To assess the OER activity of the synthesized electrocatalysts, electrochemical measurements were performed in a conventional three-electrode configuration using 1 M KOH as the electrolyte. The LSV was employed to evaluate the overpotential (η), a key metric of catalytic efficiency, at a scan rate of 5 mV s^−1^. The LSV polarization curves of CeO_2_/NF, Ce-Ni/NF, Ce-Ni-PAA_0.2_/NF, Ce-Ni-PAA_0.5_/NF, and Ce-Ni-PAA_0.8_/NF are presented in Figure 6a, with corresponding data summarized in Figure 6c. Among all investigated samples, Ce-Ni-PAA_0.5_/NF demonstrated the most outstanding OER performance, requiring an overpotential of only 366.5 mV to achieve a current density of 50 mA cm^−2^. In comparison, CeO_2_/NF, Ce-Ni/NF, Ce-Ni-PAA_0.2_/NF, and Ce-Ni-PAA_0.8_/NF exhibited higher overpotentials of 412.7, 398.8, 376.3, and 397.5 mV, respectively, under identical conditions, signifying relatively inferior catalytic activity. The remarkable performance of Ce-Ni-PAA_0.5_/NF can be ascribed to its optimized composition and the synergistic interaction between CeO_2_, NiO, and PAA, which collectively enhance the number of electrochemically active sites, facilitate rapid electron transport, and promote efficient adsorption/desorption of OER intermediates. This observation underscores the critical role of PAA in tailoring the nanostructure and boosting OER efficiency. To gain deeper insight into the reaction kinetics, Tafel slope analysis was performed, as illustrated in Figure 6b,c. The Ce-Ni-PAA_0.5_/NF catalyst exhibited the lowest Tafel slope of 93.5 mV dec^−1^, indicative of faster charge-transfer kinetics and more favorable OER dynamics. This value was significantly lower than those of CeO_2_/NF (111.9 mV dec^−1^), Ce-Ni/NF (108.9 mV dec^−1^), Ce-Ni-PAA_0.2_/NF (96.2 mV dec^−1^), and Ce-Ni-PAA_0.8_/NF (99.8 mV dec^−1^), further confirming the superior intrinsic catalytic activity of the Ce-Ni-PAA_0.5_/NF electrode.

The OER in alkaline media proceeds through a series of four well-defined mechanistic steps, each involving the transfer of electrons and participation of hydroxide ions (OH^−^) at the catalyst’s active site, typically represented as M (a transition metal center) [49,50]. A Tafel slope value of approximately 120 mV dec^−1^ is widely interpreted as an indicator that the rate-limiting process is the initial OH^−^ adsorption and electron transfer [51]:

M + OH^−^ → M-OH + e^−^ (2)

When the measured Tafel slope decreases below this, it suggests a shift in the rate-determining step toward subsequent reaction stages, such as the progressive formation of the M-O intermediate and the subsequent conversion to M-OOH:

M-OH + OH^−^ →M-O + H_2_O_(l)_ + e^−^ (3)

M-O + OH^−^ → M-OOH + e^−^ (4)

The final evolution of oxygen is accomplished by the transformation of the M-OOH species, liberating molecular oxygen and regenerating the active center:

M-OOH + OH^−^ → M + O_2(g)_ + H_2_O_(l)_ + e^−^ (5)

For the Ce-Ni-PAA_0.5_/NF catalyst, a Tafel slope of 93.5 mV dec^−1^ was observed, unequivocally pointing to the formation of the Ni-OOH species as the rate-limiting step, highlighting refined catalytic kinetics and efficient active site utilization in the overall OER cycle.

**Figure 7:**
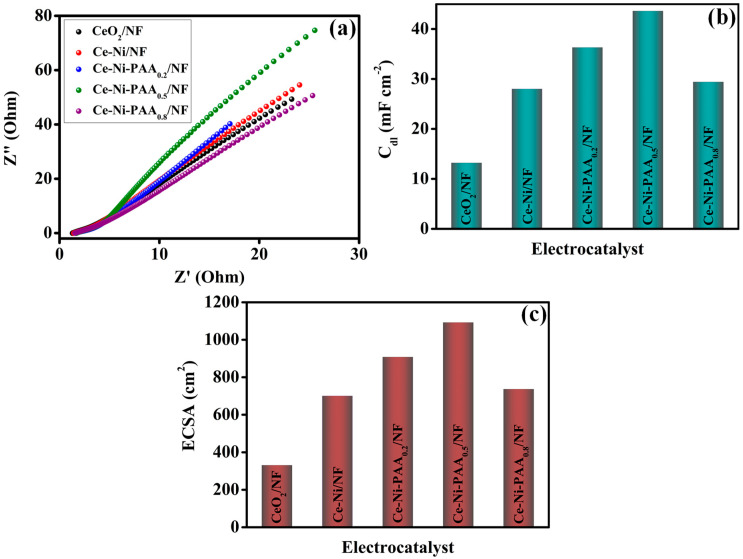
(**a**) EIS spectra, (**b**) C_dl_ and (**c**) ECSA results of all the electrocatalyst.

Electrochemical impedance spectroscopy (EIS) was employed to probe the charge transfer characteristics and interfacial resistance of the synthesized electrocatalysts under OER conditions. The Nyquist plots presented in Figure 7a clearly demonstrate that Ce-Ni-PAA_0.5_/NF exhibits the smallest semicircle diameter, indicative of the lowest charge transfer resistance (R_ct_). The Ce-Ni-PAA_0.5_/NF composite achieved the lowest R_ct_ and solution resistance (R_s_) values of 16.78 Ω and 1.42 Ω, respectively, surpassing the other tested electrocatalysts in electron transfer efficiency. In comparison, pristine CeO_2_ exhibited higher R_ct_ and slightly lower R_s_ values, measured at 27.47 Ω and 1.30 Ω, respectively. Intermediate resistance values were observed for Ce-Ni/NF (R_ct_: 25.44 Ω, R_s_: 1.554 Ω), Ce-Ni-PAA_0.2_/NF (R_ct_: 19.97 Ω, R_s_: 1.574 Ω), and Ce-Ni-PAA_0.8_/NF (R_ct_: 22.25 Ω, R_s_: 1.66 Ω). These results underscore the superior electron transfer kinetics realized in the optimally engineered Ce-Ni-PAA_0.5_/NF composite. The reduced R_ct_ of Ce-Ni-PAA_0.5_/NF reflects more efficient electron transport across the electrode-electrolyte interface, which is critical for accelerating the OER kinetics. The synergistic interaction between CeO_2_, NiO, and PAA within the hybrid architecture thus facilitates rapid interfacial charge migration, enabling superior catalytic activity. Cyclic voltammetry (CV) measurements were further performed at varying scan rates to evaluate the electrochemical double-layer capacitance (C_dl_) and estimate the electrochemically active surface area (ECSA). Figure S4 displays the CV profiles for CeO_2_/NF (Figure S4a), Ce-Ni/NF (Figure S4b), Ce-Ni-PAA_0.2_/NF (Figure S4c), Ce-Ni-PAA_0.5_/NF (Figure S4d), and Ce-Ni-PAA_0.8_/NF (Figure S4e), all of which exhibit a proportional increase in current response with increasing scan rate, confirming capacitive behavior and efficient charge storage capability. The 2C_dl_ graph is presented in Figure S5, and C_dl_ values derived from the slope of the capacitive current versus scan rate plots (Figure 7b) were determined to be 13.2, 28.0, 36.3, 43.6, and 29.4 mF cm^−2^ for CeO_2_/NF, Ce-Ni/NF, Ce-Ni-PAA_0.2_/NF, Ce-Ni-PAA_0.5_/NF, and Ce-Ni-PAA_0.8_/NF, respectively. These results directly correlate with the electrochemically accessible surface area of each electrode. Subsequently, the ECSA was estimated using the relation: ECSA = C_dl_/C_s_, where C_s_ is the specific capacitance of a flat surface in 1 M KOH (≈0.040 mF cm^−2^) [52]. As shown in Figure 7c, the calculated ECSA values were 331.0, 700.1, 907.5, 1092.3, and 736.3 cm^2^ for CeO_2_/NF, Ce-Ni/NF, Ce-Ni-PAA_0.2_/NF, Ce-Ni-PAA_0.5_/NF, and Ce-Ni-PAA_0.8_/NF, respectively. Notably, Ce-Ni-PAA_0.5_/NF exhibited the highest ECSA, signifying the presence of a significantly larger number of electrochemically active sites compared to other samples. This enhancement can be attributed to the optimal incorporation of PAA, which promotes a more uniform distribution of CeO_2_ nanoflakes and NiO nanoparticles, thereby maximizing surface exposure and active site availability. The expanded surface area collectively contributes to the superior charge transfer capability and elevated OER activity of Ce-Ni-PAA_0.5_/NF.

**Figure 8:**
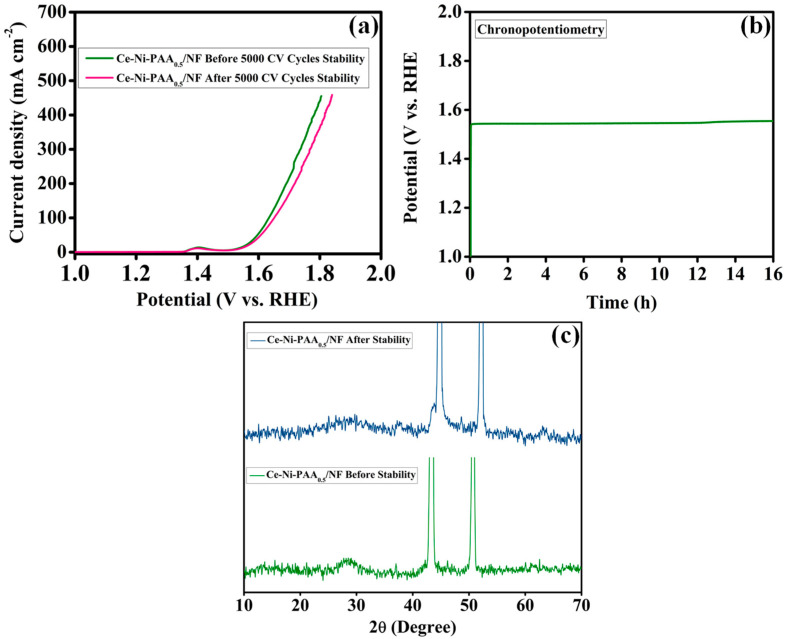
(**a**) LSV curves of Ce-Ni-PAA_0.5_/NF before and after 5000 CV cycles, (**b**) Chronopotentiometry analysis, and (**c**) After stability XRD spectra of Ce-Ni-PAA_0.5_/NF.

The long-term stability of electrocatalysts is a critical parameter for evaluating their practical viability, particularly in large-scale electrochemical energy conversion applications. To investigate the structural and electrochemical robustness of the heterostructured Ce-Ni-PAA_0.5_/NF catalyst, durability tests were conducted in 1.0 M KOH using CV and chronopotentiometry. The CV stability test was performed at a scan rate of 50 mV s^−1^ under continuous cycling to simulate extended operational conditions. As shown in Figure 8a, the LSV polarization curves recorded before and after 5000 CV cycles exhibit minimal deviation, with the overpotential measured at 50 mA cm^−2^ increasing only slightly from its initial value to 378.3 mV after cycling. This negligible performance loss highlights the excellent structural integrity and sustained electrocatalytic activity of Ce-Ni-PAA_0.5_/NF. To further validate its long-term durability, chronopotentiometric measurements were carried out at a constant current density of 10 mA cm^−2^ for 16 h (Figure 8b). The electrode displayed a stable potential response with no significant increase over the duration of the test, confirming its outstanding operational stability under continuous OER conditions. The combination of superior cycling stability and prolonged durability underscores the robustness of the Ce-Ni-PAA_0.5_/NF electrode, making it a promising candidate for practical deployment in alkaline water splitting. Figure 8c displays the XRD spectra recorded for the Ce-Ni-PAA_0.5_/NF catalyst after extended stability testing. The major diffraction peaks attributable to CeO_2_ and NiO are moderately preserved, underscoring the catalyst’s robust structural framework and retention of principal crystalline phases. Importantly, two new reflections observed at approximately 12.5° and 25.1° can be confidently indexed to the NiOOH phase, in close agreement with JCPDS card No. 00-006-0075 [53]. The formation of NiOOH is particularly advantageous, as this phase is known to significantly enhance catalytic performance in water oxidation reactions.

## 4. Conclusions

In summary, a Ce-Ni-PAA_0.5_/NF electrocatalyst was successfully fabricated via a facile electrodeposition approach, yielding a robust and hierarchically structured material with excellent catalytic performance toward the OER. Among the synthesized compositions, Ce-Ni-PAA_0.5_/NF demonstrated the most remarkable activity, achieving a low overpotential of 366.5 mV at 50 mA cm^−2^, coupled with a favorable Tafel slope and reduced charge transfer resistance, as evidenced by electrochemical impedance spectroscopy. These superior results are attributed to the synergistic interaction between CeO_2_ nanoflakes, NiO nanoparticles, and the PAA polymer network, which collectively increase the density of electrochemically active sites, facilitate efficient electron transport, and accelerate OH^−^ ion diffusion at the electrode-electrolyte interface. Furthermore, the optimized Ce-Ni-PAA_0.5_/NF exhibited a significantly enlarged ECSA and excellent long-term operational stability, confirming its potential for sustained OER catalysis under alkaline conditions. The findings underscore the critical role of PAA as a structure-directing and binding agent, enabling homogeneous distribution of active phases and promoting strong interfacial contact, thereby improving overall catalytic efficiency.

## Data Availability

The data presented in this study are available on request from the corresponding author.

## Supplementary Materials

The following supporting information can be downloaded at: https://www.mdpi.com/article/10.3390/polym17192631/s1, Figure S1: SEM micrograph images of (a1–a3) Ce-Ni-PAA0.2/NF, and (b1–b3) Ce-Ni-PAA0.8/NF; Figure S2: (a1,a2) EDAX analysis data, and (a3–a5) Elemental mapping data of Ce-Ni-PAA0.2/NF; Figure S3: (a1,a2) EDAX analysis data, and (a3–a5) Elemental mapping data of Ce-Ni-PAA0.8/NF; Figure S4: Cyclic voltammetry analysis of all the electrocatalysts at different scan rate; Figure S5: 2Cdl graph of all the electrocatalysts; Table S1: Comparison of present electrocatalyst OER performance result with other reported state-of-the-art electrocatalysts.

## References

[1] Xu Z., Wu Z.-S. (2025). Scalable production of high-performance electrocatalysts for electrochemical water splitting at large current densities. eScience.

[2] Pan D., Yu B., Tressel J., Yu S., Saravanan P., Sangoram N., Ornelas-Perez A., Bridges F., Chen S. (2025). Rapid Synthesis of Carbon-Supported Ru-RuO_2_ Heterostructures for Efficient Electrochemical Water Splitting. Adv. Sci..

[3] Lyu L.M., Chang Y.C., Li H.J., Wang P.E., Juang R.H., Lu M.Y., Li C.S., Kuo C.H. (2025). Turning the Surface Electronic Effect Over Core-Shell CoS_2_─Fe_x_Co_1_-_x_S_2_ Nanooctahedra Toward Electrochemical Water Splitting in the Alkaline Medium. Adv. Sci..

[4] Ranjith B., Gnanasekaran L., Karthika P., Rajabathar J.R., Al-Lohedan H.A., Kim W.K., Reddy V.R.M., Kapoor M., Singh S., Lavanyaj M. (2025). Enhancing the activity of transition metal-based sulfides via synergistic effects for electrochemical overall water splitting. Int. J. Hydrogen Energy.

[5] Ye S.H., Shi Z.X., Feng J.X., Tong Y.X., Li G.R. (2018). Activating CoOOH porous nanosheet arrays by partial iron substitution for efficient oxygen evolution reaction. Angew. Chem. Int. Ed..

[6] Zhao J.-W., Li C.-F., Shi Z.-X., Guan J.-L., Li G.-R. (2020). Boosting lattice oxygen oxidation of perovskite to efficiently catalyze oxygen evolution reaction by FeOOH decoration. Research.

[7] Yu J., Cao Q., Feng B., Li C., Liu J., Clark J.K., Delaunay J.-J. (2018). Insights into the efficiency and stability of Cu-based nanowires for electrocatalytic oxygen evolution. Nano Res..

[8] Song R., Wang X., Ge J. (2023). Recent progress of noble metal-based single-atom electrocatalysts for acidic oxygen evolution reaction. Curr. Opin. Electrochem..

[9] Hou L., Peng X., Lyu S., Li Z., Yang B., Zhang Q., He Q., Lei L., Hou Y. (2025). Advancements in MXene-based nanohybrids for electrochemical water splitting. Chin. Chem. Lett..

[10] Lin S., Mandavkar R., Habib M.A., Dristy S.A., Joni M.H., Jeong J.-H., Lee J. (2025). Fabrication of Ru-doped CuMnBP micro cluster electrocatalyst with high efficiency and stability for electrochemical water splitting application at the industrial-level current density. J. Colloid Interface Sci..

[11] Vazhayil A., Vazhayal L., Thomas J., Thomas N. (2021). A comprehensive review on the recent developments in transition metal-based electrocatalysts for oxygen evolution reaction. Appl. Surf. Sci. Adv..

[12] Han L., Dong S., Wang E. (2016). Transition-metal (Co, Ni, and Fe)-based electrocatalysts for the water oxidation reaction. Adv. Mater..

[13] Liu F., Feng Z., Zhang X., Cui L., Liu J. (2023). One-step achievement of Fe-doped and interfacial Ru nanoclusters co-engineered Ni (OH)_2_ electrocatalyst on Ni foam for promoted oxygen evolution reaction. J. Colloid Interface Sci..

[14] Li Y., Bao X., Chen D., Wang Z., Dewangan N., Li M., Xu Z., Wang J., Kawi S., Zhong Q. (2019). A minireview on nickel-based heterogeneous electrocatalysts for water splitting. ChemCatChem.

[15] Kitiphatpiboon N., Chen M., Li X., Liu C., Li S., Wang J., Peng S., Abudula A., Guan G. (2022). Heterointerface engineering of Ni_3_S_2_@ NiCo-LDH core-shell structure for efficient oxygen evolution reaction under intermittent conditions. Electrochim. Acta.

[16] Kim J., Kim H., Han G.H., Hong S., Park J., Bang J., Kim S.Y., Ahn S.H. (2022). Electrodeposition: An efficient method to fabricate self-supported electrodes for electrochemical energy conversion systems. Exploration.

[17] Abebe E.M., Ujihara M. (2022). Simultaneous electrodeposition of ternary metal oxide nanocomposites for high-efficiency supercapacitor applications. ACS Omega.

[18] Miao M., Duan H., Luo J., Wang X. (2022). Recent progress and prospect of electrodeposition-type catalysts in carbon dioxide reduction utilizations. Mater. Adv..

[19] Minakshi M., Aughterson R., Sharma P., Sunda A.P., Ariga K., Shrestha L.K. (2025). Micelle-Assisted Electrodeposition of γ-MnO_2_ on Lead Anodes: Structural and Electrochemical Insights. ChemNanoMat.

[20] Minakshi M., Higley S., Baur C., Mitchell D.R., Jones R.T., Fichtner M. (2019). Calcined chicken eggshell electrode for battery and supercapacitor applications. RSC Adv..

[21] Biswal A., Panda P.K., Acharya A.N., Mohapatra S., Swain N., Tripathy B.C., Jiang Z.-T., Minakshi Sundaram M. (2020). Role of additives in electrochemical deposition of ternary metal oxide microspheres for supercapacitor applications. Acs Omega.

[22] Somacescu S., Osiceanu P., Moreno J.M.C., Navarrete L., Serra J.M. (2013). Mesoporous nanocomposite sensors based on Sn_1−x_Ce_x_O_2−δ_ metastable solid solution with high percentage of Ce^3+^ valence state for selective detection of H_2_ and CO. Microporous Mesoporous Mater..

[23] Gao W., Wen D., Ho J., Qu Y. (2019). Incorporation of rare earth elements with transition metal–based materials for electrocatalysis: A review for recent progress. Mater. Today Chem..

[24] Tian L., Liu H., Zhang B., Liu Y., Lv S., Pang L., Li J. (2021). Ni and CeO_2_ nanoparticles anchored on cicada-wing-like nitrogen-doped porous carbon as bifunctional catalysts for water splitting. ACS Appl. Nano Mater..

[25] Patel K.B., Mariyaselvakumar M., Vyas G., Chaudhari J.C., Patidar R., Srinivasan K., Srivastava D.N., Bhadu G.R. (2024). Nickel oxide doped ceria nanoparticles (NiO@ CeO_2_) for boosting oxygen evolution reaction and enhancing stability. Appl. Surf. Sci..

[26] Ghosh D., Pradhan D. (2023). Effect of cooperative redox property and oxygen vacancies on bifunctional OER and HER activities of solvothermally synthesized CeO_2_/CuO composites. Langmuir.

[27] Yan Y., Xia B.Y., Zhao B., Wang X. (2016). A review on noble-metal-free bifunctional heterogeneous catalysts for overall electrochemical water splitting. J. Mater. Chem. A.

[28] Qin C., Fan A., Ren D., Luan C., Yang J., Liu Y., Zhang X., Dai X., Wang M. (2019). Amorphous NiMS (M: Co, Fe or Mn) holey nanosheets derived from crystal phase transition for enhanced oxygen evolution in water splitting. Electrochim. Acta.

[29] Manzoor S., Mansha M., Ali S., Altaf F., Shams A., Khan S.A. (2025). Facile synthesis of porous multiple hydroxyl and amine polymer@ NiO composite for stable and efficient electrochemical water splitting. J. Power Sources.

[30] Bibi H., Mansoor M.A., Asghar M.A., Ahmad Z., Numan A., Haider A. (2025). Facile hydrothermal synthesis of highly durable binary and ternary cobalt nickel copper oxides for high-performance oxygen evolution reaction. Int. J. Hydrogen Energy.

[31] Lin C., Zhao Y., Zhang H., Xie S., Li Y.-F., Li X., Jiang Z., Liu Z.-P. (2018). Accelerated active phase transformation of NiO powered by Pt single atoms for enhanced oxygen evolution reaction. Chem. Sci..

[32] Xin X., Zhang Y., Wang R., Wang Y., Guo P., Li X. (2023). Hydrovoltaic effect-enhanced photocatalysis by polyacrylic acid/cobaltous oxide–nitrogen doped carbon system for efficient photocatalytic water splitting. Nat. Commun..

[33] Wang F., Liang W.J., Jian J.X., Li C.B., Chen B., Tung C.H., Wu L.Z. (2013). Exceptional poly (acrylic acid)-based artificial [FeFe]-hydrogenases for photocatalytic H_2_ production in water. Angew. Chem. Int. Ed..

[34] Biswal A., Sethy P.K., Swain S.K. (2021). Change in orientation of polyacrylic acid and chitosan networks by imprintment of gold nanoparticles. Polym. Plast. Technol. Mater..

[35] Amano Y., Nakagawa Y., Ohta S., Ito T. (2018). Ion-responsive fluorescence resonance energy transfer between grafted polyacrylic acid arms of star block copolymers. Polymer.

[36] Hackett A.J., Malmström J., Travas-Sejdic J. (2019). Grafting poly (acrylic acid) from PEDOT to control the deposition and growth of platinum nanoparticles for enhanced electrocatalytic hydrogen evolution. ACS Appl. Energy Mater..

[37] Guo Y., Tang J., Henzie J., Jiang B., Xia W., Chen T., Bando Y., Kang Y.-M., Hossain M.S.A., Sugahara Y. (2020). Mesoporous iron-doped MoS_2_/CoMo_2_S_4_ heterostructures through organic–metal cooperative interactions on spherical micelles for electrochemical water splitting. ACS Nano.

[38] Panneerselvam P., Singh C., Jayaraj S.K., Doulassiramane T., Padmanaban R., Samal A.K., Mohan S., Jadhav A.H. (2024). Unveiling the impact of oxygen vacancies in engineered bimetallic oxides for enhanced oxygen evolution reaction: Insights from experimental and theoretical approaches. J. Mater. Chem. A.

[39] Ding X., Jiang R., Wu J., Xing M., Qiao Z., Zeng X., Wang S., Cao D. (2023). Ni_3_N–CeO_2_ heterostructure bifunctional catalysts for electrochemical water splitting. Adv. Funct. Mater..

[40] Bhosale M., Baby N., Magdum S.S., Murugan N., Kim Y.A., Thangarasu S., Oh T.-H. (2024). Hierarchical nanoassembly of Ni_3_S_2_-MoS_2_ interconnected with CeO_2_ as a highly remarkable hybrid electrocatalyst for enhancing water oxidation and energy storage. J. Energy Storage.

[41] Tang C., Zhong L., Xiong R., Xiao Y., Cheng B., Lei S. (2023). Regulable in-situ autoredox for anchoring synergistic Ni/NiO nanoparticles on reduced graphene oxide with boosted alkaline electrocatalytic oxygen evolution. J. Colloid Interface Sci..

[42] Bhosale M., Thangarasu S., Magdum S.S., Jeong C., Oh T.-H. (2024). Enhancing the electrocatalytic performance of vanadium oxide by interface interaction with rGO and NiO nanostructures for electrochemical water oxidation. Int. J. Hydrogen Energy.

[43] Zhang R., Qiao Q., Yu B., Zhang Z., Wang J., Li S., Liu Y., Yuan H., Luo J., Wang Y. (2025). Formation of CeO_2_/NiO composites with intergrowth superstructures for photocatalytic CO_2_ reduction. Nano Res..

[44] Zhao L., Wang L., Zhou J., Xu H., Wang Z., Liu Y., Liao X., Nie M. (2025). CeO_2_ facilitates electron transfer at the Fe-Ni_2_ P heterointerface, enhancing the overall process of water splitting. J. Mater. Chem. A.

[45] Huang Y., Ding X., Huang B., Xie Z. (2024). CeO_2_-decorated Fe-doped Ni_2_P nanosheets for efficient electrocatalytic overall water splitting at high current densities. J. Alloys Compd..

[46] Baby N., Thangarasu S., Murugan N., Kim Y.A., Oh T.-H. (2025). MOF derived Fe_3_O_4_/NiO decorated rGO-BN for efficient electrochemical water splitting. Int. J. Hydrogen Energy.

[47] Sha W., Song Y., Liu P., Wang J., Xu B., Feng X., Guo J. (2022). Constructing multiple heterostructures on nickel oxide using rare-earth oxide and nickel as efficient bifunctional electrocatalysts for overall water splitting. ChemCatChem.

[48] Ghosh D., Manikanta Kumar M., Raj C.R., Pradhan D. (2022). Bifunctional catalytic activity of solvothermally synthesized CeO_2_ nanosphere/NiO nanoflake nanocomposites. ACS Appl. Energy Mater..

[49] Li C., Baek J.-B. (2021). The promise of hydrogen production from alkaline anion exchange membrane electrolyzers. Nano Energy.

[50] Yao S., Wei H., Zhang Y., Zhang X., Wang Y., Liu J., Tan H.H., Xie T., Wu Y. (2021). Controlled growth of porous oxygen-deficient NiCo_2_ O_4_ nanobelts as high-efficiency electrocatalysts for oxygen evolution reaction. Catal. Sci. Technol..

[51] Kauffman D.R., Alfonso D., Tafen D.N., Lekse J., Wang C., Deng X., Lee J., Jang H., Lee J.-S., Kumar S. (2016). Electrocatalytic oxygen evolution with an atomically precise nickel catalyst. ACS Catal..

[52] Pan U.N., Singh T.I., Paudel D.R., Gudal C.C., Kim N.H., Lee J.H. (2020). Covalent doping of Ni and P on 1T-enriched MoS_2_ bifunctional 2D-nanostructures with active basal planes and expanded interlayers boosts electrocatalytic water splitting. J. Mater. Chem. A.

[53] Chen K., Cao Y.-H., Yadav S., Kim G.-C., Han Z., Wang W., Zhang W.-J., Dao V., Lee I.-H. (2023). Electronic structure reconfiguration of nickel–cobalt layered double hydroxide nanoflakes via engineered heteroatom and oxygen-vacancies defect for efficient electrochemical water splitting. Chem. Eng. J..

