# Supplementary material for: Electropolymerized PAA as a Functional Matrix for CeO2-NiO Hybrid Electrocatalysts for Efficient Water Oxidation
Source: Polymers (Basel). 2025 Sep 28;17(19):2631. doi: 10.3390/polym17192631 (PMC12526815; doi:10.3390/polym17192631)
Supplement: Supplementary file 1 [file polymers-17-02631-s001.zip › polymers-3897588-supplementary.pdf]

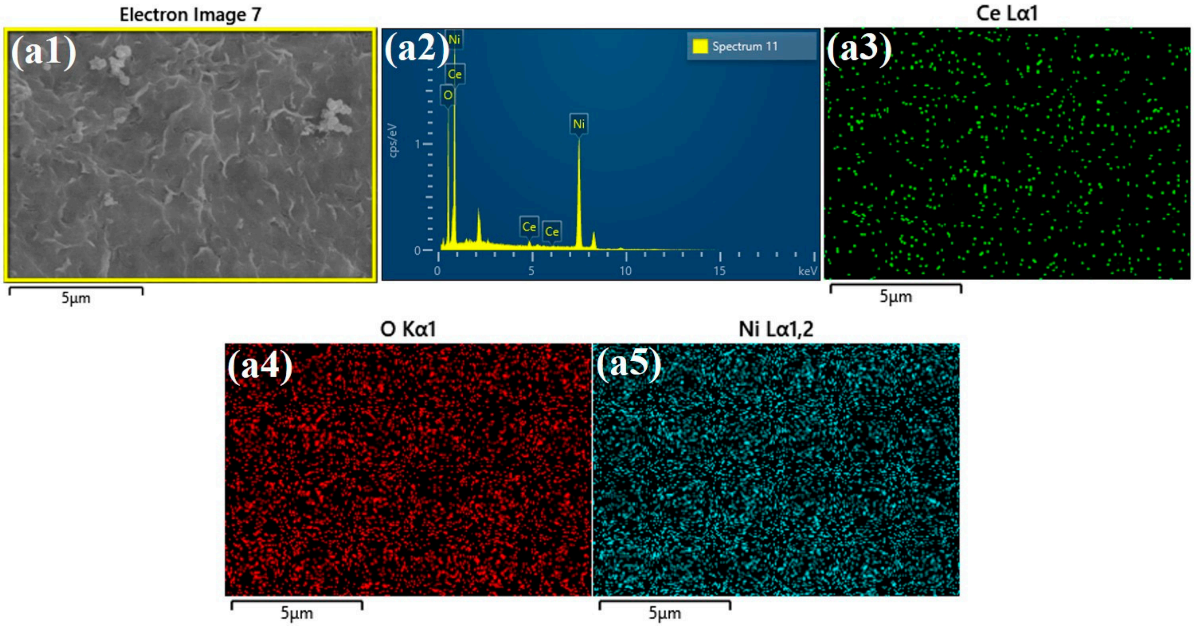

Electropolymerized PAA as a Functional Matrix for CeO_2-NiO Hybrid Electrocatalysts for Efficient Water Oxidation.

Mrunal Bhosale[1], Pritam J. Morankar[1], Yeonsu Lee[1], Hajin Seo[1], and Chan-Wook Jeon[1]*

[1]*School of Chemical Engineering, Yeungnam University, 280 Daehak-ro, Gyeongsan 712-749, Republic of Korea.*

Contact Information

Corresponding author: Prof. Chan-Wook Jeon - cwjeon@ynu.ac.kr

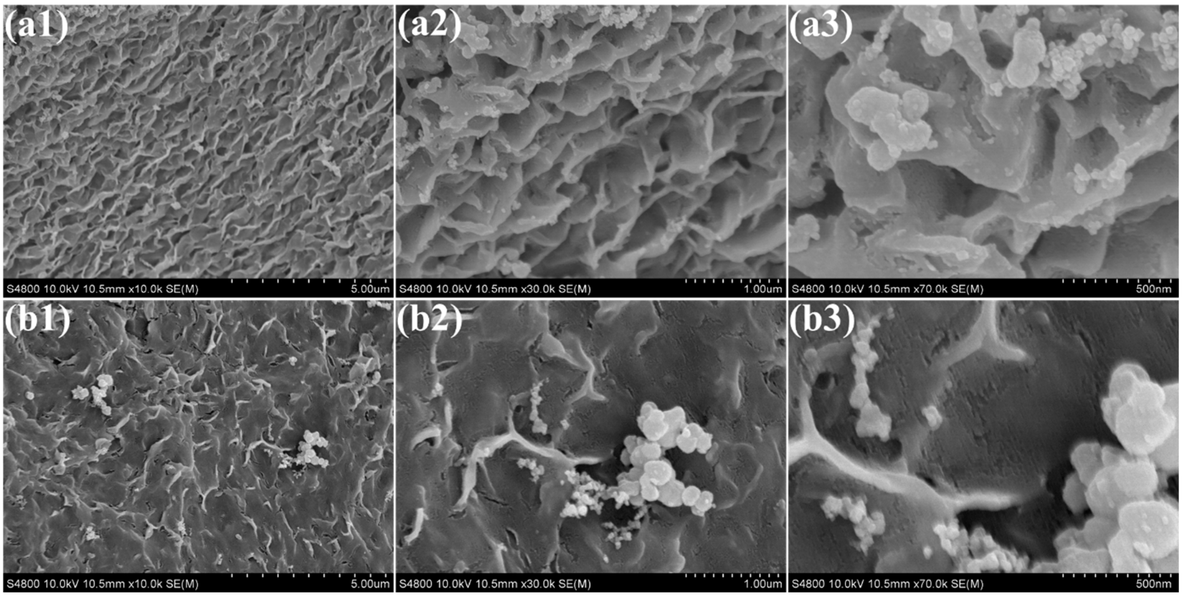

Figure S1: SEM micrograph images of (a1-a3) Ce-Ni-$PAA_{0.2}$/NF, and (b1-b3) Ce-Ni-$PAA_{0.8}$/NF.

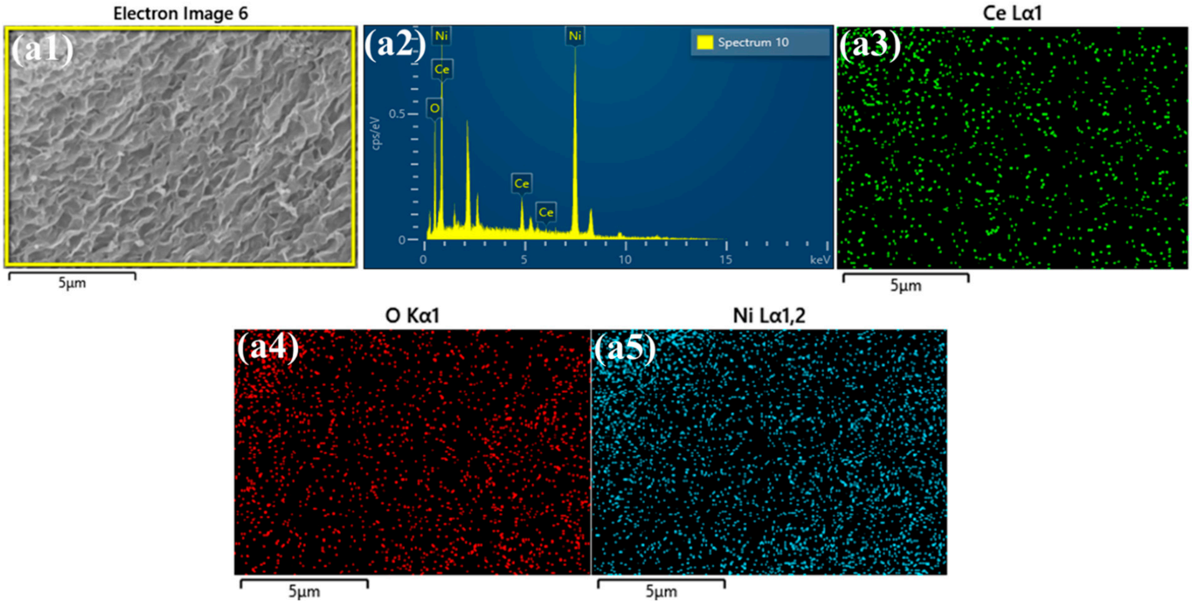

Figure S2: (a1-a2) EDAX analysis data, and (a3-a5) Elemental mapping data of Ce-Ni-$PAA_{0.2}$/NF.

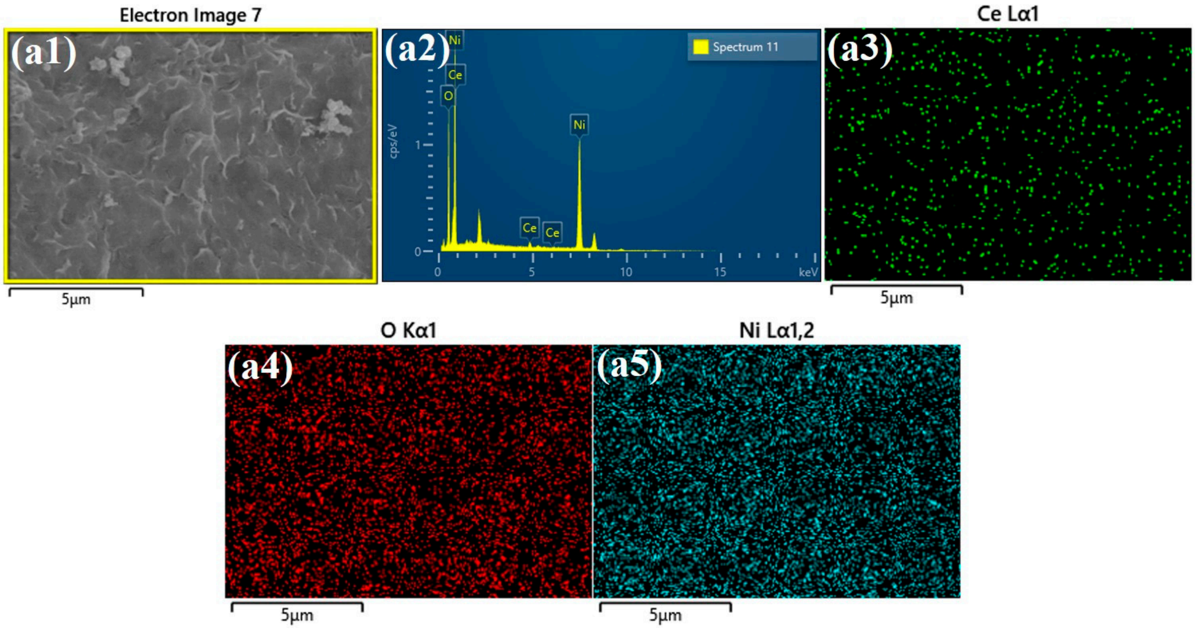

Figure S3: (a1,a2) EDAX analysis data, and (a3-a5) Elemental mapping data of Ce-Ni-$PAA_{0.8}$/NF.

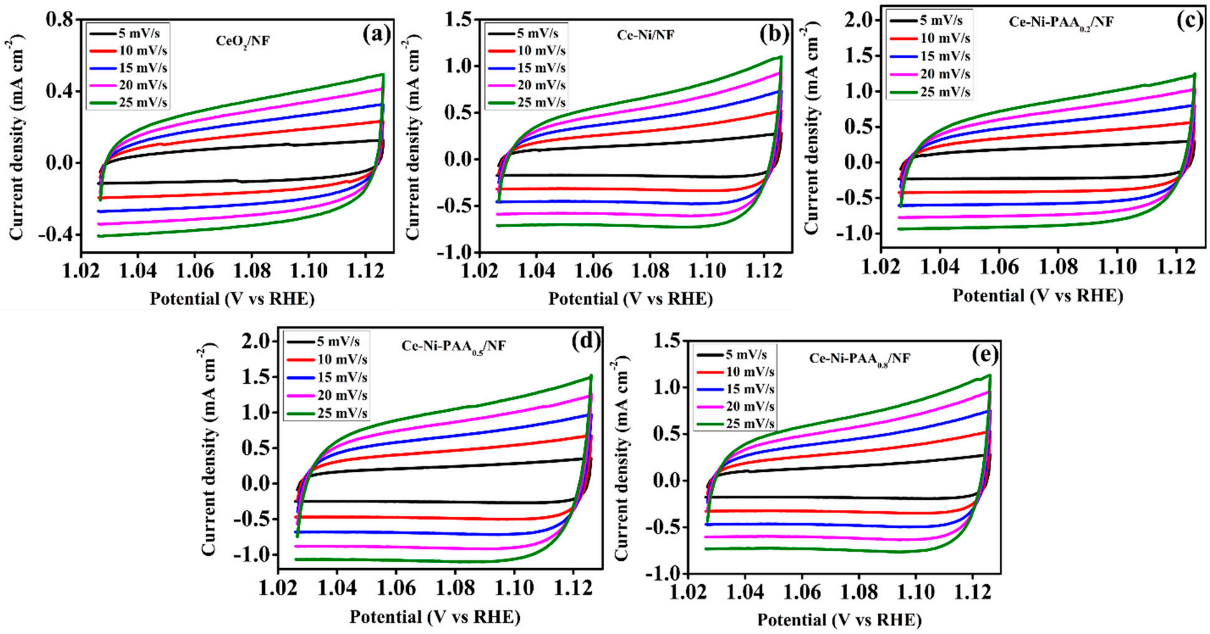

Figure S4: Cyclic voltammetry analysis of all the electrocatalysts at different scan rate.

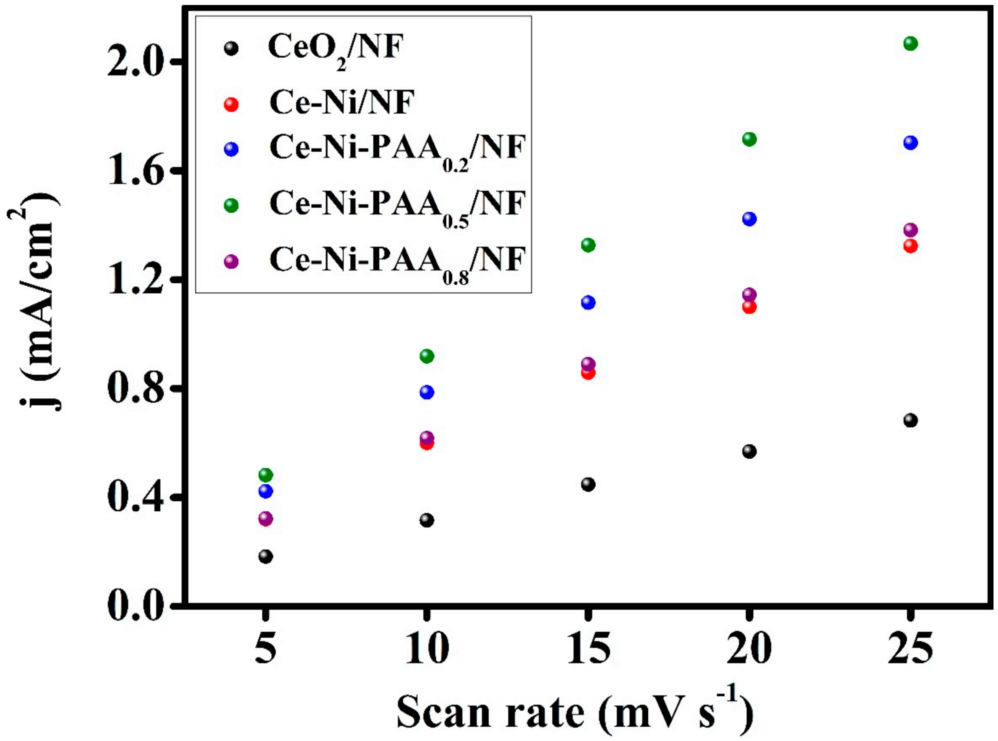

Figure S5: $2C_{dl}$ graph of all the electrocatalysts.

Table S1: Comparison of present electrocatalyst OER performance result with other reported state-of-the-art electrocatalysts.

Material	Overpotential	Electrolyte	Ref.
$NiCo_2O_4$-CD	390 mV@50 mA/cm^2	1M KOH	[1]
NiP_x	390@50 mA/cm^2	1M KOH	[2]
CuO from Cu-EA	475@10 mA/cm^2	1M KOH	[3]
Polyethyleneimine functionalized graphene oxide	350 mV@50 mA/cm^2	1M KOH	[4]
IrO_2-Ta_2O_5	326 mV@10 mA/cm^2	1M KOH	[5]
Mn-modified RuO_2	293 mV@10 mA/cm^2	1 M KOH	[6]
carbon-supported Ru/RuO_2	300 mV@10 mA/cm^2	1 M KOH	[7]

References:

[1] Kundu, A., Ryplida, B. and Park, S.Y., 2020. Carbon dots integrated $NiCo_2O_4$ hierarchical nanoneedle arrays supported on Ni foam as efficient and stable electrode for hydrogen and oxygen evolution reactions. Electroanalysis, 32(9), pp.2090-2100.

[2] Kodinzev, I., Lobinsky, A. and Tolstoy, V., 2019, January. Successive ionic layer deposition of NiPx nanolayers on the surface of nickel foam and their electrocatalytic properties for oxygen evolution reaction upon water splitting in alkaline medium. In AIP Conference Proceedings (Vol. 2064, No. 1, p. 030005). AIP Publishing LLC.

[3] Liu, X., Cui, S., Qian, M., Sun, Z. and Du, P., 2016. In situ generated highly active copper oxide catalysts for the oxygen evolution reaction at low overpotential in alkaline solutions. Chemical communications, 52(32), pp.5546-5549.

[4] Ghouri, Z.K., Elsaid, K., Badreldin, A., Nasef, M.M., Jusoh, N.W.C. and Abdel-Wahab, A., 2021. Enhanced oxygen evolution reaction on polyethyleneimine functionalized graphene oxide in alkaline medium. Molecular Catalysis, 516, p.111960.

[5] Li, H., Pan, Y., Wu, L., He, R., Qin, Z., Luo, S., Yang, L. and Zeng, J., 2023. Highly active and stable IrO2 and IrO2–Ta2O5 catalysts for oxygen evolution reaction. International Journal of Hydrogen Energy, 48(67), pp.26021-26031.

[6] Xue, Z.H., Mahmood, J., Shang, Y., Li, G., Kim, S.J., Han, Y. and Yavuz, C.T., 2025. Simple and Scalable Introduction of Single-Atom Mn on RuO2 Electrocatalysts for Oxygen Evolution Reaction with Long-Term Activity and Stability. Journal of the American Chemical Society, 147(21), pp.17839-17848.

[7] Chen, J.L., Feng, S.Y., Lu, C.J. and Huang, J.F., 2023. Janus Ru/RuO2 nano-boomerangs on carbon as pH-universal electrocatalysts with bifunctional activity toward the hydrogen/oxygen evolution reaction. Chemical Engineering Journal, 468, p.143761.
